# Case of Empyema: With Remarks

**Published:** 1833-10

**Authors:** William Stroud

**Affiliations:** one of the Presidents of the Society; Physician to the Northern Dispensary, &c.


*Case of Empyema: with Remarks.* Communicated to the Harveian Society, by WILLIAM STROUD, M.D. one of the Presidents of the Society; Physician to the Northern Dispensary, &c.

A COLLECTION in any of the thoracic sacs of liquid, whether serous, or purulent, the product of inflammation, and the cause of additional disorder, is a morbid condition of much interest, and of not unfrequent occurrence. Such a collection may either originate within the part itself, or may be derived from without. Vomicæ of the lungs, for example, not unfrequently burst into the pleural sac; and abscesses in the parietes of the chest sometimes, although rarely, take a similar course. In either of these cases, it is usually to be expected that the rupture of the membrane will lead to its inflammation, and to a rapid increase of the liquid introduced.

The following is an example of simple pleuritis on the right side of the chest, attended with a copious effusion of liquid, at first serous, but ultimately purulent, which, by its powerful and continued pressure, occasioned obliteration of the lung; and, notwithstanding the temporary relief afforded by tapping, at length destroyed life by exhaustion.

Abraham B***, aged thirteen years, was placed under my care, as a patient of the Northern Dispensary, March 31, 1832. When five years old, he suffered an injury of the forehead, in consequence of which his head was for some time liable to a sort of oppression, which was spontaneously relieved by occasional bleedings at the nose. Although of rather delicate constitution, he subsequently enjoyed good health until within the last two months; during which, owing, as is supposed, to his having overstrained his back by carrying heavy burdens, he has been seriously ill, and, since the last month, has been confined to his bed. His symptoms are at present as follows: Irregular fever, with dryness of the skin, and pain in the back of the head; which has lately, however, been relieved by the application of a few leeches to the temples. Pulse at the wrist about 140, very small, and weak, so as scarcely to admit of being counted. Tongue clean, and moist, with much thirst, and little appetite. Bowels rather lax, dejections dark coloured, and hard: urine natural. Sleep disturbed. Mental faculties unimpaired. He has much disorder of respiration, frequent palpitation of the heart, troublesome cough, and a white, viscid expectoration tinged with blood. He constantly lies on the right side, in which posture he is tolerably tranquil; but, on assuming the erect posture, he is often seized with nausea, and sometimes vomits a watery liquid. If he attempts to lie on the left side, the cough and difficulty of breathing are so much increased that he is soon induced to resume his usual position. On carefully examining the chest, a remarkable contrast is soon perceived between its two sides. On the left side the respiration is puerile, and a clear, tympanic sound is elicited by percussion. On the right the respiration is nearly inaudible, and the sound yielded on percussion is perfectly dull. Unless between the shoulders, the spinal column bears handling well; but almost the whole abdomen, except the right iliac region, is painful on pressure.

*Prescriptions.* Hirud. vi. scrob. cord. Vesicatorium inter scapulas. Ol. Ricini fʒij. primo mane, prout opus fuerit. Aquæ puræ f℥vi.; Cetacei: Sacch. purificat. *aa* ʒij.; Pulv. Acaciæ gummi, ʒiv.; Tinct. Digital. m. xx.; Tinct. Camph. comp. fʒiij. Sumatur f℥i. ter, vel quater indies.

April 6. The leeches drew a very large quantity of thick, black blood; the blister was not applied. Bowels open; dejections dark coloured, and scybalous. He is free from pain, and his cough is somewhat relieved; but he has much palpitation of the heart, is still affected with nausea on attempting to sit up, and his sleep is disturbed. On further examination, the right side of his chest is now observed to be somewhat protuberant.

*Prescriptions.* Pil. Hydrarg. gr. iij. cum Ext. Hyoscyam. gr. iv. omni vesp. Rep. med. antea præscripta.

April 11. Dejections rather more natural: urine sufficiently copious. Abdomen bears pressure well: cough continues relieved: sleeps better, and has less palpitation of the heart: pulse very frequent, small, and feeble: countenance good: point of the tongue rather red, with prominent papillæ: skin dry, with dark-coloured, adherent sordes, not easily removed by washing. The whole right side of the chest is now found to be decidedly, and equably protuberant, yielding a dull sound on percussion, and transmitting little, or no respiratory murmur. Pressure between the ribs, or on the right hypochondrium, gives pain.

*Prescriptions.* Aquæ puræ f℥vi.; Potass. Nitrat. ʒi.; Pulv. Acaciæ gummi, ʒiij.; Tinct. Gentian. comp. fʒiij.; Tinct. Camphor. comp. fʒiv. Sumatur f℥i. ter in dies. Repet. Pil. Hydrarg. cum Extract. Hyoscyam. omni vespere, etiam Ol. Ricini primo mane, prout opus fuerit.

April 16. The symptoms continuing nearly the same, and the indications of a large collection of liquid in the right pleural sac being sufficiently manifest, it was determined, with the concurrence of my colleagues, Dr. Roget and Mr. Skey, to try the effect of puncturing, and gradually evacuating, the right side of the chest. To the indications previously mentioned, Mr. Skey, after a separate investigation, added the following: Extrusion of the heart towards the left side; an obscure sense of fluctuation, on using careful percussion between the ribs of the right side, and enlargement of the intercostal spaces. Dr. Thomas Davies, who had the kindness to give his assistance, suggested two more, namely: On measuring the circumference of the chest, the right side was found to exceed the left by nearly two inches; and, on inserting a needle into the right pleural sac, a drop, or two of serous fluid exuded.

All doubt respecting the nature of the case having been thus removed, the right side of the chest was now punctured by Mr. Skey, with a moderate-sized trocar, a little below the inferior angle of the scapula, and about a pint of transparent, yellow serum was immediately extracted. A catheter of elastic gum, furnished with a plug, was afterwards introduced into the pleural sac, and retained there by plaster, and bandage.

*Prescriptions* continued, with the exception of substituting

Tincturæ Hyoscyam. fʒi. in the place of Tincturæ Camphor. comp. fʒiv.

April 19. On this day, as on each preceding one, eight or ten ounces of yellow serum were extracted by Mr. Skey from the right pleural sac. During the first two or three days the irritation produced by the instrument excited occasional nausea. The boy is somewhat better, can lie on either side, has scarcely any cough, and sleeps tolerably well.

The respiratory murmur can now be heard in the upper part of the right chest. Pulse at the wrist small, feeble, and scarcely to be counted: tongue thinly coated with yellowish mucus; little thirst or appetite; no pain of abdomen: bowels moderately open; dejections whitish: urine scanty and turbid.

*Prescriptions.* Hydrarg. Submuriat. Sacch. purificat. *aa* gr. ij.; Pulv. Scammon. gr. iv. Fiat pulvis, primo mane sumendus, prout opus fuerit. Aquæ fervent. Oj.; Gummi Tragacanth. ʒi. Fiat potio mucilaginosa, cui frigefactæ adjicr. Tinct. Hyocyam., Tinct. Scillæ, *aa* fʒi.; Tinct. Cinch. comp. fʒiv. Sumatur f℥i. ter in dies. Repet. omni vespere Pil. Hydrarg. et Extract. Hyoscyam.

April 23. The serum daily extracted from the right pleural sac has gradually become denser, and more albuminous. That drawn yesterday, and to-day, was observed to contain a portion of greyish pus, which subsided to the bottom of the vessel. Yesterday morning, the left side of the abdomen was attacked with severe pain, accompanied by frequent vomiting of mucous liquid; but these symptoms have since abated. The pulse is, however, very frequent, and rather stronger: skin dry and hot: tongue whitish, with red edges, and thirst. Except to-day, the bowels have been sufficiently open, and the dejections better coloured. The urine is copious, and reddish. The pil. hydrarg. was omitted yesterday evening, and ten grains of extract. hyoscyam. were given at once, yet the sleep was disturbed.

*Prescriptions.* Hirud. iv. abdom. sinistro. Ol. Ricin. fʒij. vel. iij. primo mane, prout opus fuerit. Extract. Hyoscyam. gr. v. omni vesp. Aquæ puræ f℥vj; Cetacei, Sacch. purificat. *aa* ʒij.; Pulv. Acaciæ gummi ʒiv.; Tinct. Hyoscyam. fʒiss.; Sumatur f℥i. ter, vel quater indies.

April 26. The leeches were not applied; and, on account of the increasing debility of the patient, the following tonic potion was this day substituted for the Mist. Cetacei, &c.; namely, Infus. Rosæ. f℥viij.; Acid. Sulph. dilut. fʒss.; Sulph. Quinin. gr. x.; Sumatur f℥i. ter in dies. The elastic gum catheter, which hitherto had been constantly retained in the right pleural sac, was yesterday withdrawn by Mr. Skey. The proportion of pus in the liquid discharged had been gradually increasing. The whole quantity of liquid drawn off during the interval of ten days, from April 16 to 25, inclusive, was nearly a gallon; of which twenty-one ounces were extracted on the 20th, and eighteen ounces on the 25th; the previous day having been the only one on which none was evacuated.

April 28. The boy has slight hectic fever, with progressive emaciation, and debility, but sleeps well, without the aid of hyoscyamus, and can lie easily on either side. He has little cough, and no pain, except in the scrob. cord. when pressed. Pulse in the radial artery 134, feeble, and scarcely to be counted: tongue natural, some thirst, little appetite: bowels rather confined, dejections still whitish: urine high-coloured, and sufficiently copious: skin dry, and slightly yellow, with tenaciously adhering sordes. The respiratory murmur is very obscure in the right side of the chest, and less distinct than formerly in the left side.

*Prescriptions.* Ol. Ricin. f℥ij. vel iij.; aut Pulv. Scamm. cum Hyd. submur. gr. viij. primo mane, prout opus fuerit. Pil. Hydrarg. gr. v. om. vesp.; Aquæ puræ f℥iv.; Liq. Ammon. Acetat. f℥ij.; Pulv. Acaciæ gummi ʒiij.; Tinct. Hyoscyam. fʒi.; Tinct. Gent. comp. fʒiv.; Sumatur f℥i. ter in dies.

30. The orifice in the right side of the chest this day spontaneously discharged a large quantity of fetid pus. The respiratory murmur is again puerile on the left side of the chest, and slightly audible in the upper part of the right side, but scarcely perceptible below. The scrob. cord. is still somewhat painful on pressure. The dejections, and urine, are more natural.

*Prescriptions* continued.

May 5. Yellow pus of extreme fetor is freely discharged from the pectoral orifice: owing, perhaps, to this fetor, the boy vomited a little to-day. His symptoms are, otherwise, chiefly negative. He has scarcely any cough, or fever, sleeps well, and can lie on either side. Pulse about 134: urine dark-coloured, but dejections nearly natural.

*Prescriptions* continued.

13. Yellow pus flows freely from the pectoral orifice; and the respiratory murmur can now be heard, in a slight degree, as low as the middle of the right side. The boy becomes gradually weaker, is consequently unwilling to sit up in bed, and yesterday evening was in a state approaching to fainting. His gums have never yet been affected by mercury, and his medicines are not regularly taken. He sleeps well, has occasional nausea, and is free from pain. Pulse 120, and rather stronger: countenance good: bowels lax; dejections rather dark-coloured: urine nearly natural.

*Prescriptions.* Infus. Cuspariæ f℥vi.; Acid. Sulphur. dilut. fʒi.; Sulphat. Quinin. gr. vi.; Tinct. Opii m. xx.; Tinct. Zingiber. fʒij.; Sumatur f℥i. ter in dies.

17. With a view to produce adhesion, or change of action in the diseased membrane, Mr. Skey endeavoured, two days since, to inject into the right pleural sac a solution of sulphate of zinc; but, owing to the difficulty of the process, and the weakness of the patient, the attempt was unsuccessful. The boy is gradually wasting, and sinking from simple exhaustion, without fever. He takes little solid food, but does not refuse wine, or porter. Much thin, fetid pus flows at intervals from the pectoral orifice, and the breath exhales a similar fetor. The respiration is occasionally short and frequent, but he has little cough. Pulse 120, and tolerably strong: tongue red: bowels sufficiently open: urine dark-coloured. During sleep the eyelids are half open, with the pupils turned upwards, and he sometimes talks, or wakes in a fright.

*Prescriptions.* Aquæ puræ f℥vi.; Aluminis fʒi.; Tinct. Opii, m. xx.; Tinct. Catechu fʒiv.; Sumatur f℥i. ter, vel quater in dies. Sulphat. Ferri; Sulphat. Zinc; Sulphat. Quinin. *aa* gr. xij.; Confect. Ros. canin. q. s. Fiant Pil. xij.; quarum sumatur i. ter, vel quater in dies.

20. After an interval of three days, a quantity of extremely fetid pus, preceded by a little blood, issued this day from the pectoral orifice; and the patient's chamber, as well as an adjacent room, are infected with its gangrenous odour. While the pus was confined, the cough was renewed, and was attended with difficulty of breathing, with nausea, and at times with vomiting. The boy is progressively sinking from simple debility, with little fever, and scarcely any thirst, or appetite. He takes white wine, but refuses porter, lies only on the right side, and sleeps pretty well. His left leg has lately been painful, but he is, otherwise, without pain; the pulse at the wrist can scarcely be counted; the mental faculties are free from disorder; the bowels and urine are natural, except that the dejections are blackish, from the action, probably, of the sulphate of iron. His skin, which has hitherto been constantly dry, and sordid, is now more moist and perspirable.

*Prescriptions.* Rep. Pilulæ tonicæ.

24. After a second interval of three days, another eruption of yellow and exceedingly fetid pus, mixed with a little blood, took place from the pectoral orifice. During this interval, as during the former one, the cough, nausea, and difficulty of breathing, were troublesome; but, on the discharge of the matter, all these symptoms subsided. Owing, apparently, to the fallacious excitement which sometimes precedes death, he seems much better to-day, complains only of a little pain in the left leg, and has a desire for acids.

*Prescriptions.* Aquæ puræ f℥vi.; Acid. Sulph. dilut. fʒi.; Sulph. Quinin. gr. x.; Pulv. Acaciæ gummi ʒiij.; Tinct. Opii m. xv.; Tinct. Gent. comp. fʒiv.; Sumatur f℥i. ter in dies. Pulv. Ipecac. comp. gr. v. hora somni.

26. After the last visit the boy rapidly declined, and died between three and four o'clock this morning; about four months from the date of his first attack, during the latter half of which, alone, he was placed under my care. In the afternoon the body was inspected by Mr. Skey, from whom I obtained the following account of the appearances observed by him.

*Post-mortem Appearances.* On the right side of the chest the costal pleura was, at its upper part, connected with the pulmonary pleura by several thick bands of adhesion. With this exception, the right pleural sac was open, enormously dilated, and lined by false membrane. It contained from three to four quarts of thin, whey-like, puriform liquid, of an extremely fetid odour, with a thicker and yellower portion lying at the bottom. By the pressure of this accumulated liquid, the diaphragm on the same side had been pushed downwards, so that the liver extended a little below the umbilicus. The right lung was condensed, but flabby, of a greyish blue colour, and about the size of the fist; its function having evidently been at length completely abolished. A small portion of the root of each lung was carnified, or converted into a red, fibrous substance resembling muscle. In other respects, the left lung, like the heart, was in a healthy state. The mesenteric glands were pale, and somewhat enlarged; but the abdominal organs were otherwise sound.

Remarks. From the case above related, the following remarks relative to the symptoms, the diagnosis, and the treatment of empyema, may naturally be deduced.

*On the Symptoms of Empyema.* The first circumstance deserving of notice is the occurrence of intense pleuritis without pain; which, in this instance, seems to have been almost entirely absent from first to last; although, at an advanced period, the sensibility of the diseased pleura was sufficiently manifest, from the pain occasioned by pressure between the ribs of the affected side, as likewise by the introduction of the needle, and the trocar. Pain must not, therefore, as formerly, be regarded as an essential character of acute inflammation of serous membranes, of which, although undoubtedly a frequent, it is by no means an inseparable attendant. To ascertain the conditions which determine its presence, or absence, would be an interesting, but difficult task. The ultimate seat of pain must be placed in the nerves, since the most acute pain may occur when no other parts are materially affected; whilst, on the other hand, when these alone are disabled, or divided, all pain is at an end. But it is not every morbid condition, even of nerves themselves, which is productive of pain; since, as in some cases of caries of the teeth, the nerves of parts, which at other times are extremely sensitive, may participate in their gradual ulceration and destruction, without the slightest pain being experienced. That the inflammation of nerves is a powerful cause of pain is abundantly proved by numerous observations; but the fugitive and transient character of some severe pains shows that inflammation is not essential to their existence. The conclusion seems, therefore, to be, that pain depends on some undefined state of irritation, that is, of increased and disordered action in nerves, which is freely communicable to their centre. Of the more remote causes of pain, pressure or tension is one of the most efficient; but even this is neither an absolute, nor an universal cause; since, as is evident in the case now under consideration, pressure on one entire lung to the extent of obliteration was unattended with pain.

A second subject for consideration is the gradual transition, under certain circumstances, of serum into pus; or, to speak more correctly, of albuminous into purulent secretion. That the qualities of exhaled or secreted liquids are in exact relation to the kind and degree of the action whence they are derived, is sufficiently manifest from the phenomena of catarrh, of diarrhœa, and of ulcers. In proportion to the progressive intensity of the local action in each case, the discharge, from being simple and watery, becomes loaded with saline or animal matter, proceeding from gelatine, and mucous, to albumen, fibrine, and ultimately to blood itself. In the subject of the present history, when the chest was first punctured, the liquid evacuated was transparent, inodorous, slightly glutinous, of a pale yellow colour, and, in short, purely albuminous; but, within a few days afterwards, pus began to be added. It was easily recognised by its opacity, incoherence, separation from the serum, and subsidence to the bottom of the vessel; and it daily increased, both in quantity and in acrimony, until, at length, the liquid became perfectly purulent, and intolerably fetid. To this unfavorable change the puncture itself may, possibly, have contributed, since, in the corresponding operation of tapping the abdomen, the same cause is often sufficient to induce or increase peritoneal inflammation; owing to which the dropsical accumulation proceeds more rapidly, and each successive puncture becomes more painful. But, as the secretion of pus did not take place until after an interval of some days, it is more probable that the elastic gum catheter retained in the pleural sac, and replaced when it occasionally escaped; or else atmospheric air, which, during inspiration, could scarcely fail to be sometimes admitted through the external aperture, was the cause of that increased and morbid action, to which the formation of pus must ultimately be ascribed. The noxious influence exercised by atmospheric air, and more especially by its oxygen, on surfaces ill adapted to sustain its impression, although not without limits and exceptions, is sufficiently proved by daily observation. One of the most familiar illustrations of this influence is furnished by burns and scalds, in which, while every other condition remains the same, the pain is uniformly and rapidly aggravated by the admission of atmospheric air, and relieved by its exclusion, whether effected by mechanical means, such as cotton-wool, the incrustation of nitrate of silver, &c., or by chemical ones, such as the oil of turpentine, and other disoxygenating substances. Numerous facts concur to show that a peculiar mode, or a higher degree of inflammatory irritation, thus induced, will convert albuminous into purulent secretion, and will give rise to putrescence and fetor, where none would otherwise exist.

A third topic for inquiry is suggested by the extremely dry, and unperspirable state of the skin, throughout the greater part of the complaint; until, in common with other parts, its texture and action seemed to soften, and relax, on the immediate approach of death. During health, and even in many diseases, free perspiration, and the perpetual abrasion of the cuticle by exercise and friction, preserve the external surface of the body in a comparatively clean and polished state; but, under that interruption of the cutaneous secretion which occurs in various disorders, and more especially in those of children, accidental sordes, or vitiated cuticle, of a dusky colour, is found to adhere so tenaciously, that neither by ablution nor by mechanical agency, can it without much difficulty be detached. This condition, immediately owing to a deficiency of exhalation, and perhaps also to an increase of absorption, is probably produced by the remote influence, through the medium of the nervous system, of some diseased internal organ, which is often suffering from the opposite state of excessive effusion.

A fourth subject of attention is the remarkable agency of this, and of similar thoracic complaints, on the abdominal functions. In consequence, perhaps, of the downward pressure of the liquid in the pleural sac, as likewise of the diminution of respiration, and of the impeded return of venous blood, slight symptoms of inflammation, irritation, or congestion, occasionally take place in the abdomen; and particularly in the liver and kidneys, as is denoted by the morbid alteration of their respective excretions; but the occurrence of the same symptoms in empyema, when occupying the left pleura, is sufficient to show that mere mechanical pressure on the liver is neither the only, nor the principal cause of such symptoms. Owing to the strong reciprocal influence of the several parts of the living body, and chiefly of those co-operating in a common and important function, such as that of nutrition, irritation is often extensively communicated from one part to another, and sometimes with a salutary and compensating effect, which used formerly to be dignified by the appellation of the *vis medicatrix naturæ.* On this, as on other occasions, the author has frequently observed the striking influence of pressure, or of other irritation, originally affecting the pleural surface of the diaphragm, in exciting nausea and vomiting. In the progress of the complaint above related, it will be remembered that when, owing to temporary obstruction of the external aperture, the accumulation of liquid in the pleural sac was to a certain extent increased, nausea and vomiting were not less obviously induced than cough and dyspnœa; and that, on an eruption of pus taking place, all these symptoms immediately and simultaneously subsided. The peculiar constitution, and important relations of the diaphragm are calculated to prevent any surprise at such effects, although the precise manner in which they are generated may be open to further inquiry.

Lastly, in considering the symptoms of empyema, an interesting comparison may be instituted between the character of simple thoracic inflammation, and that of phthisis. In pleuritis, for example, the symptoms of debility, and of exhaustion predominate; while in phthisis, those of hectic fever, and of universal irritation, are usually more conspicuous; and the capacity of the sound portions of the lungs to maintain in some measure the function of the entire organ seems to be greater. The more decided and deleterious influence of phthisis on the general health, is probably owing to its coming more immediately and actively in contact with the pulmonary nerves, more especially those belonging to the ganglionic system, which, under the agency of pleuritis and its results, are either protected, or destroyed. In like manner, in the progress of phthisis itself, all the symptoms become strikingly aggravated, whenever, by the supervention of inflammation, or of suppuration, the parenchyma of the lungs is more deeply and more violently irritated.

*On the Diagnosis.* In noticing the diagnosis of empyema, it is impossible to overlook the immense superiority of the indications afforded by the modern resources of auscultation, and percussion over those resulting from the remote, and general symptoms. During the early part of the case here considered, the latter means alone were employed ; and owing to the absence of pain in the chest, and of urgent dyspnœa, the complaint was regarded by a very respectable practitioner as simple fever, attended with a determination of blood to the head. Nor could it well have been otherwise, since daily experience proves that, without extensive and accurate investigation, aided by direct physical indications, the highest degree of professional knowledge and talent is often inadequate to determine the nature and seat of disease; whereas, with these advantages, a very moderate share of intelligence and information are equal to the task. In the present case, when the mechanical exploration of the chest was at length employed, the evidence furnished by the opposite sounds elicited from its two sides was prompt and decisive ; and, had this mode of inquiry been employed at the commencement, it might, perhaps, have led to a happier termination. A circumstance may here be briefly noticed, which on reflection might have been anticipated, namely, that the impossibility of hearing the respiratory murmur, when there is much liquid collected in the pleural sac, does not depend on any deficiency in the liquid as a conductor of sound, but on the compression which it exercises on the subjacent lung. This is proved by a fact, which the author has repeatedly observed, namely, that when an abscess is seated in the parietes of the chest, without encroaching on its cavity, the respiratory murmur can be distinctly heard through the contained pus. Decisive as is, in such cases, the evidence of sound, there are some persons of a peculiarly cautious and inflexible frame of mind, whom it fails to convince, and who, strangely enough, are disposed to ascribe their difficulty of conviction to superiority of intelligence. In favor of these persons, it is fortunate that there exists an additional, and an irresistible means of elucidation, in the needle, or minute trocar employed by Dr. T. Davies, an expedient which, in other respects, can rarely be required; but which, in determining the presence or absence of a liquid in the chest, is happily beyond the reach of contradiction.

*On the Treatment of Empyema.* In this part of the subject, the principal topic for consideration, is the propriety and utility of puncturing and evacuating the pleural sac. When the diagnosis is clearly ascertained, there is a strong inducement to perform this operation, on account of the facility with which a morbid and otherwise permanent collection of liquid may thereby be removed. But, unless there is either an evident tendency to spontaneous perforation, denoted by slight inflammation and œdema of the adjacent integuments, or much danger of suffocation, or of some other serious evil resulting from its omission, experience seems to be rather unfavorable to its use. The proportion of successful and of unsuccessful cases is, at least, nearly equal, showing the importance of investigating and avoiding all the sources of failure. If the operation is deemed requisite, the sooner it is performed the better, on account of the rapid tendency of the compressed lung to become condensed and obliterated; a circumstance which furnishes an additional argument in favour of early and accurate diagnosis. The contrast between what may be termed the dry and the humid forms of pleuritis, is, in this respect, remarkable. When new or false membranes alone are the product of inflammation, they have a slow but powerful tendency to contract, and thereby to constrict the parts with which they are connected, until both the pleura and the whole side are much diminished; whereas, under liquid effusion, the pleural sac becomes progressively enlarged, and the lung alone is compressed.

When puncture of the chest is to be performed, it should probably be executed with all the precautions usually observed in opening psoas, or lumbar abscess, namely, by a small oblique and valvular orifice, which is afterwards to be closed, and healed as soon as possible; and as in tapping the abdomen, a fresh aperture may be made from time to time, as occasion requires. The admission of atmospheric air, and all other sources of irritation, should be studiously avoided; and, with such care and circumspection, the operation, if seasonably practised, under favourable circumstances, may be expected, not indeed to restore an organ already destroyed, but to relieve urgent symptoms, and to prevent further mischief.

